# Physical-layer data of IRIDIUM satellites broadcast messages

**DOI:** 10.1016/j.dib.2023.108905

**Published:** 2023-01-13

**Authors:** Gabriele Oligeri, Savio Sciancalepore, Roberto Di Pietro

**Affiliations:** a Division of Information and Computing Technology, College of Science and Engineering, Hamad Bin Khalifa University, Doha 34110, Qatar; b Department of Mathematics and Computer Science, Eindhoven University of Technology, Eindhoven 5612, the Netherlands

**Keywords:** Iq samples, Iridium ring alert, Software-defined radio, Wireless communications, Satellite communications

## Abstract

Physical-layer information associated with wireless communications is a trove of data, that can be leveraged by several research communities, e.g., networking and security. Indeed, such information (IQ samples) represents the signal at the very beginning of the receiver chain, just after the demodulation, and they embed valuable information about both the channel and the transmitter, which can be used for several purposes, e.g., protocol design, network performance analysis, and transmitter fingerprinting.

In this paper, we present the data of a measurement campaign targeting the messages of the IRIDIUM satellite constellation. The resulting dataset has been collected throughout a measurement period of about 2 months and it comprises +3.8 M IRIDIUM Ring Alert (IRA) packets—the cleartext packets broadcasted by the IRIDIUM satellites. Our dataset includes several pieces of information included in the IRA packets, i.e., the reception timestamp, position of the transmitting satellite on the ground, satellite ID, beam ID, etc. Moreover, for each packet, we also collected the corresponding raw IQ samples, for a total of +7.6B data.

We believe that the amount of collected data, the duration of the measurement campaign, and the information included herein, will be valuable assets for the research community.

**Specifications Table**

**Table:** 

**Subject**	Computer Science
**Specific subject area**	Physical-layer data acquisition (IQ samples) of broadcast messages emitted by the satellites of the IRIDIUM constellation.
**Type of data**	Table
**How the data were acquired**	The data have been acquired by resorting to state-of-art equipment, i.e., a Software-Defined Radio USRP X310 featuring a daughterboard UBX160 connected to an IRIDIUM active antenna RST740, and the GNU Radio open-source software, modified on-purpose to log the data. The antenna was deployed on the second floor out of the window of a Villa for a total measurement period of about 2 months.
**Data format**	Raw
**Description of data collection**	The measurement campaign has been carried out in two periods, i.e., from Aug. 12th to Sept. 10th, 2020, and Sept. 11th to Oct. 9th, 2020. The dataset includes a total of 3840,907 entries (IRIDIUM broadcast messages), and 7628,604,575 IQ samples.
**Data source location**	Institution: Hamad bin Khalifa University City/Town/Region: Doha Country: Qatar Latitude and longitude (and GPS coordinates, if possible) for collected samples/data: [25.3238903, 51.4187532]
**Data accessibility**	Repository name: Mendeley Data Data identification number: 10.17632/xcxspv8c2r.2 Direct URL to data: https://data.mendeley.com/datasets/xcxspv8c2r/2

## Value of the Data

• This dataset constitutes amassive collection of physical-layer information of messages captured from the existing satellite constellation (IRIDIUM).
• This dataset includes information from the physical layer of the satellite radio link exposing details and peculiarities that are not visible to higher layers, i.e., in-phase (I) and quadrature (Q) samples associated with the transmitted bits.
• IQ samples are used by several research communities, such as networking (study, design and implementation of effective modulation/demodulation techniques) and security (e.g. transducer authentication at the physical-layer).
• The duration of the measurement campaign (almost 2 months) and the amount of collected information (broadcast messages from all the IRIDIUM satellites) represent valuable information for both academia and industry.

## 1 Objective

The objective of our measurement campaign was to collect physical-layer information from an operating satellite network (IRIDIUM), to prove with real experimental data, the feasibility of physical-layer authentication of satellite transducers [1]. This dataset [2] and the associated analysis [1] provide a comprehensive study of the physical layer of satellite networks.

## 2 Data Description

The dataset [2] is constituted by 2 files:

• 1208–1009_20_parsed.txt (7.5 GB)

Non-stop measurements of IRIDIUM Ring Alert (IRA) messages received at our location, acquired from Aug. 12th to Sept. 10th, 2020. The number of table entries are 2060,289.

• 1109–0910_20_parsed.txt (6.7 GB)

Non-stop measurements of IRIDIUM Ring Alert (IRA) messages received at our location, acquired from Sept. 11th to Oct. 9th, 2020. The number of table entries are 1780,618.

Each file can be imported in any data analysis tool for further visualization and analysis. For instance, it is possible to import the files in MatLab using the following command:

$$d=readtable\left(<path\_to\_the\_file>,`ReadVariableNames^{\prime },false\right)$$

The associated data structure in the workspace (d) is constituted by the following fields:

• *Var1: Timestamp*. Epoch unix timestamp in microseconds (µs).
• *Var2: Timestamp*. Timestamp of the local machine, in milliseconds (ms).
• *Var2: Satellite ID*. Unique identifier of the transmitting satellite. It can take the following values [2 3 4 5 6 7 8 9 13 16 17 18 22 23 24 25 26 28 29 30 33 36 38 39 40 42 43 44 46 48 49 50 51 57 65 67 68 69 71 72 73 74 77 78 79 81 82 85 87 88 89 90 92 93 94 96 99 103 104 107 109 110 111 112 114 115], for a total of 66 satellites.
• *Var3: Beam ID*. Identifier associated with the beam (antenna array) of the considered satellite. The beam ID spans between 0 and 48, for a total of 49 IDs.
• *Var4: Latitude*. Current latitude of the satellite at ground, in degrees.
• *Var5: Longitude*. Current longitude of the satellite at ground, in degrees.
• *Var6: Satellite altitude*. Estimated current altitude of the satellite (Km). We found this information might be inconsistent.
• *Var7: Confidence index*. Indicator of the confidence about the correctness of the received bits, as provided by the gr-iridium module of GNU Radio, between a min value of 0 and a max value of 100.
• *Var8: Frequency*. Specific carrier frequency of the channel where the information is received.
• *Var9: IQ samples*. Raw IQ samples, reported in the form of a cell array of complex numbers, i.e., {‘(a1+jb1), (a2+jb2). ....’}.

.

**Fig. 1:**
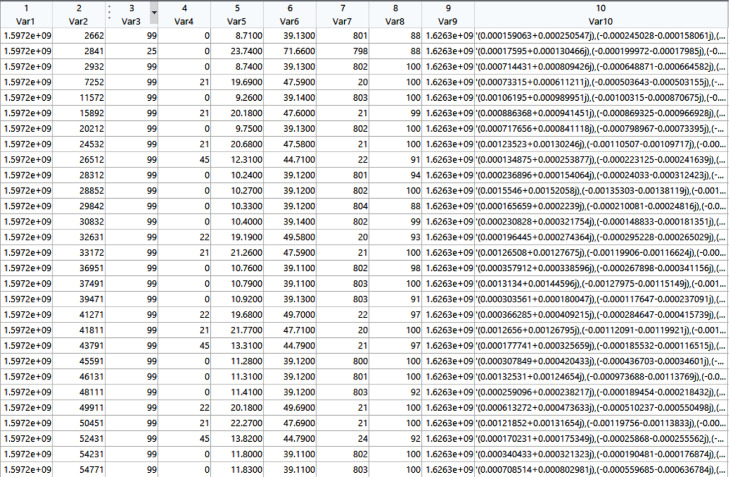
A snapshot of the MATLAB data structure associated with the dataset.

Fig. 1. Shows an excerpt of the data structure in the MATLAB workspace.

## 3 Experimental Design, Materials and Methods

**Fig. 2:**
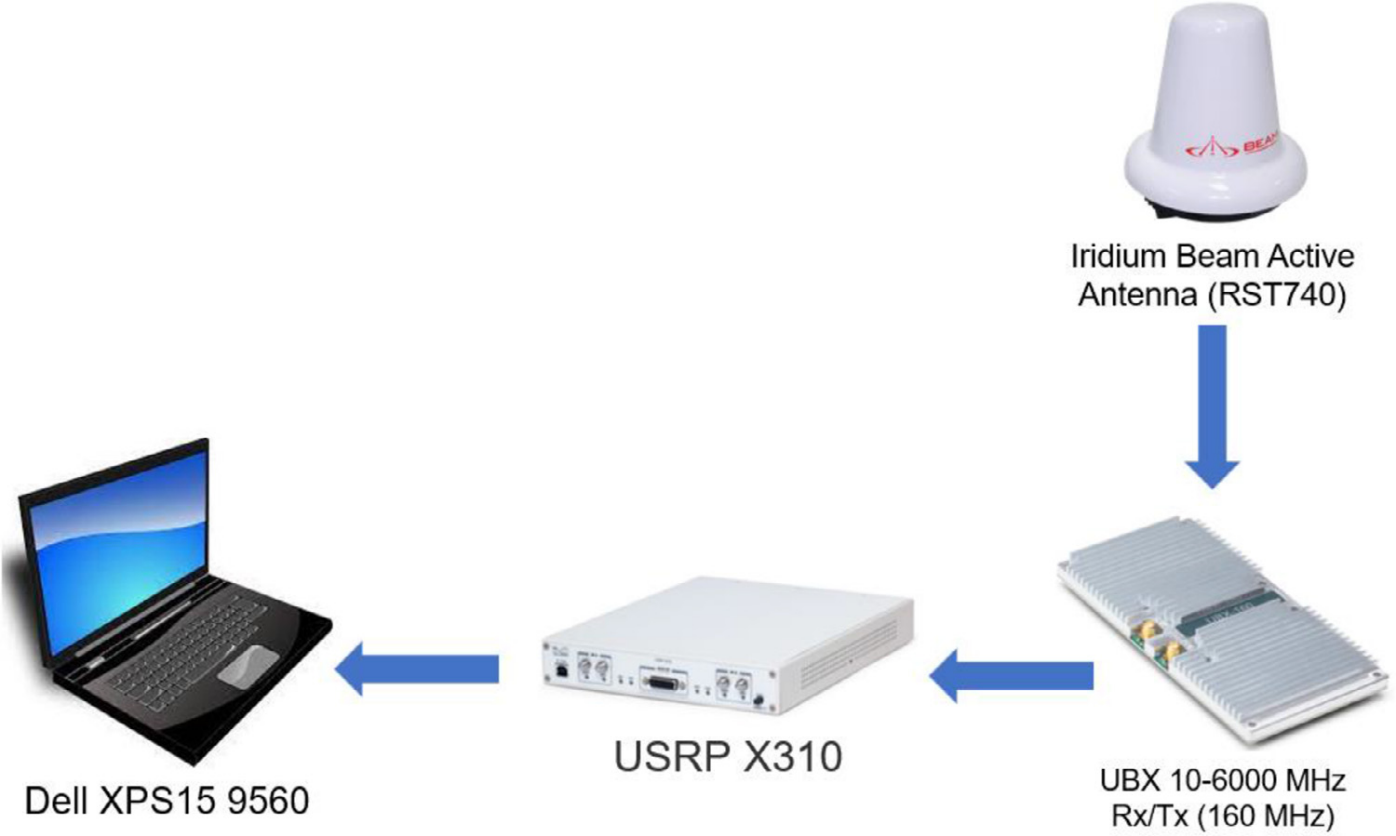
Hardware setup for data acquisition, modified from [1].

Hardware set-up**.** The hardware setup used for data acquisition is depicted in Fig. 2. The data have been acquired by means of a Software-Defined Radio (SDR) USRP X310, featuring a UBX-160 daughterboard and an IRIDIUM Beam active antenna model RST740. The SDR was connected via Ethernet to a laptop Dell XPS15 9560 (32GB RAM, 8 Intel Cores i7700HQ – 2.8 GHz), running the software described below.

We acquired the data by holding the IRIDIUM antenna out of a window on the second floor of a villa located in Education City – Doha (Qatar) at GPS coordinates [25.3238903, 51.4187532], facing the direction 220°.

Note that researchers interested in reproducing our data collection should use only the IRIDIUM Beam active antenna model RST740. We attempted multiple times to gather the data using different sub-optimal general-purpose antennas, but all our attempts ended up either in no reception of IRIDIUM packets or very high packet losses. Moreover, the receiving antenna should be placed outside, possibly under a clear open sky. Our experimentation showed that the conditions described above maximize the reception range and minimize packet losses.

### 3.1 Software Set-up

To orchestrate the data collection process and exchange data with the SDR board, we used the well-known GNU Radio Development Toolkit [3]. GNU Radio is a free and open-source software, providing blocks for interfacing with any SDRs, performing data processing, and implementing software processes to run on SDRs. For this data acquisition, we used GNU Radio version 3.8, and we leveraged the processing functions provided by the extension module gr-iridium, specifically dedicated to the decoding of IRIDIUM packets [4]. Within the blocks provided by the gr-iridium tool, we enabled the logging of the information of our interest by adding new code within the function "iridium_qpsk_demod_cpp_impl.cc", immediately after the filtering of the received bitstream through the Phased Locked Loop (PLL), at line 408 of the corresponding library. Then, through a modification of the script "iridium-parser.py" of the gr-iridium module, we enabled the logging of additional information compared to the one already collected by gr-iridium, i.e., the reception timestamp of the IRIDIUM messages on the SDR and the raw I-Q samples corresponding to the decoded packet.

As part of our data processing chain, we also used the software tool iridium-toolkit, i.e., a collection of tools to parse IRIDIUM messages [5]. Specifically, we used the parser in the iridium-toolkit tool to obtain only the IRIDIUM Ring Alert (IRA) messages. Then, through a custom-built shell script, we extracted from the IRA messages all the information contained in our dataset, as described above (timestamps, satellite identifier, beam identifier, latitude of the satellite, longitude of the satellite, altitude of the satellite, confidence index of the message, reception frequency, and corresponding raw I-Q samples).

The collected data should be considered as raw, i.e., neither additional filtering nor analysis has been performed over the data collected during the collection campaign, besides the processing strictly required for extracting physical-layer information from the wireless channel.

## Ethics Statements

Hereby, the authors consciously assure that for this manuscript the following is fulfilled: this material is the authors' own original work, which has not been previously published elsewhere; the paper is not currently being considered for publication elsewhere; the paper reflects the authors' own research and analysis in a truthful and complete manner; the paper properly credits the meaningful contributions of co-authors and co-researchers.; all sources used are properly disclosed (correct citation); all authors have been personally and actively involved in substantial work leading to the paper, and will take public responsibility for its content.

## CRediT authorship contribution statement

**Gabriele Oligeri:** Conceptualization, Methodology, Software, Validation, Investigation, Resources, Data curation, Writing – original draft. **Savio Sciancalepore:** Conceptualization, Methodology, Software, Validation, Investigation, Resources, Data curation, Writing – original draft. **Roberto Di Pietro:** Conceptualization, Validation, Writing – review & editing, Supervision, Funding acquisition.

## Declaration of Competing Interest

The authors declare that they have no known competing financial interests or personal relationships that could have appeared to influence the work reported in this paper.

## Data Availability

Physical layer data acquisition of IRIDIUM satellites broadcast messages (Original data) (Mendeley Data)
